# Sudden elevation of liver enzymes in a 64-year-old patient: a case report

**DOI:** 10.1186/1757-1626-2-205

**Published:** 2009-11-18

**Authors:** Marcus Wiedmann, Constanze Müller, Hartmut Lobeck, Katharina Wölke

**Affiliations:** 1 Department of Internal Medicine II, University of Leipzig, Germany; 2 Department of Internal Medicine I, St. Mary's Hospital, Berlin, Germany; 3 Office for Internal Medicine and Cardiology, Berlin-Wilmersdorf, Germany; 4 Institute of Pathology, Ernst von Bergmann Hospital, Potsdam, Germany; 5 Institute of Pathology, St. Gertrauden-Hospital, Berlin, Germany

## Abstract

Eradication of Helicobacter pylori usually consists of a 7-day course of triple therapy including metronidazole or amoxicillin plus clarithromycin plus a proton pump inhibitor. We report about a rare adverse event of Hp eradication in a patient with moderate chronic and moderate active pangastritis. Shortly after the end of treatment cholestatic hepatitis occurred which was most likely related to clarithromycin, perhaps enhanced by amoxicillin. Since liver dysfunction was self-limited, no further treatment was required. In summary, clinicians should be aware about the presented rare adverse event of Helicobacter pylori eradication treatment for a close monitoring of those patients and rapid management of acute liver failure.

## Introduction

According to the current DGVS (German Association of Gastroenterologists) S3-guidelines eradication of Helicobacter pylori (Hp) is indicated in patients with gastric or duodenal ulcer and gastric marginal zone B-cell lymphoma of MALT-type (mucosa-associated lymphoid tissue). It is facultative in patients with dyspepsia (following upper GI endoscopy), chronic asymptomatic Hp-associated gastritis, Mënëtrier's disease, idiopathic thrombocytopenic purpura, and lymphocytic gastritis [1]. A 7-day course of triple therapy is recommended including metronidazole or amoxicillin plus clarithromycin plus a proton pump inhibitor (PPI). Common adverse events of clarithromycin are nausea, vomiting, abdominal tenderness, diarrhea, (very) rare adverse events are crampy abdominal pain, pseudomembranous colitis, acute pancreatitis, elvated liver enzymes, interstitial nephritis, hypersensitivity reaction, "Torsades de pointes" tachycardia, tinnitus, hearing loss, dizziness, confusion, anxiety, sleeplessness, nightmares, hallucinations, psychosis, headache, hypoglycemia, leukopenia, and thrombocytoenia (according to patient information leaflet). Amoxicillin my induce diarrhea, gastritis, stomatitis, nausea, vomiting, glossitis, darkening of the tongue, enterocolitis, pseudomembranous colitis, hypersensitivity reaction, mild elevation of ASAT, anemia, thrombocytopenia, eosinophilia, and agranulocytosis (according to patient information leaflet).

## Case presentation

**Figure 1:**
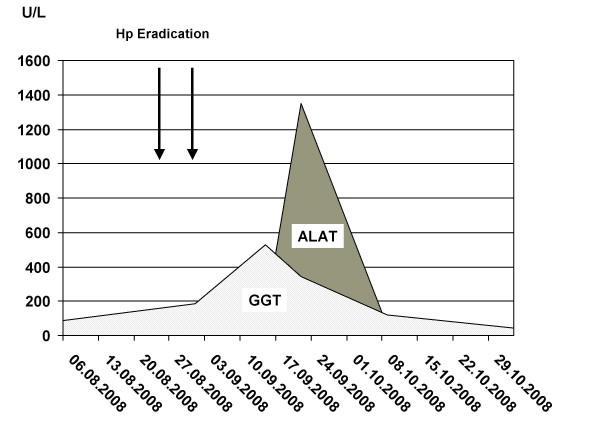
**Follow-up of serum ALAT and GGT levels in a 64 year old man with drug-induced liver injury**.

In August 2008, a 64-year-old Caucasian male German presented at our outpatient department with complaints of nausea, knife-like pain in the right upper quadrant of the abdomen, which occurred after the intake of food, and additional globus pharyngis feeling. He had a past medical history of arterial hypertension, hyperuricemia, benign paroxysmal positional vertigo, and was allergic to diclofenac. The list of drugs he was taken included 6 mg betahistine q.d., 150 mg allopurinol q.d., 25 mg carvedilol q.d., 80 mg valsartan b.i.d., and 25 mg hydrochlorothiazide q.d. Initial laboratory investigation showed increased levels for ASAT [67 U/L (normal ≤35)], ALAT [67 U/L (normal ≤45)], GGT [87 U/L (normal ≤55)], and bilirubin [22 μmol/L (normal < 20)] (Figure 1).

**Figure 2:**
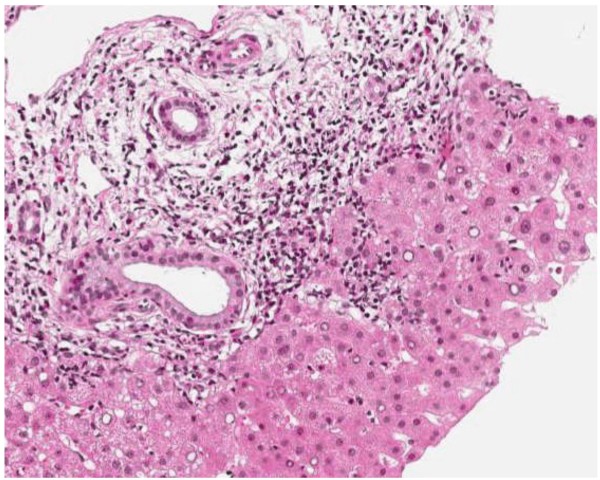
**Liver biopsy shows portal inflammation on hematoxylin-eosin stain (magnification ×100)**.

**Figure 3:**
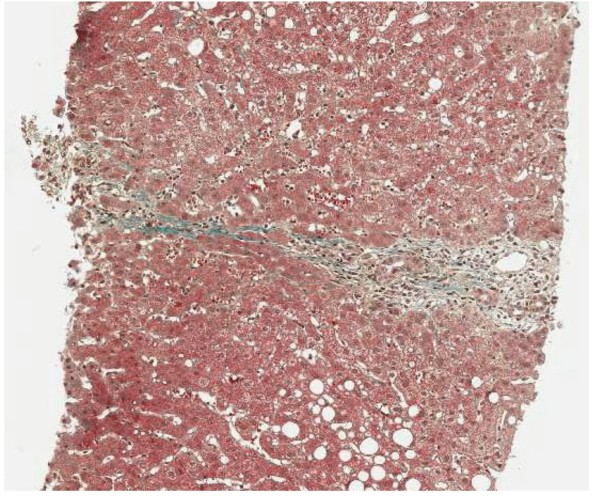
**Liver biopsy shows beginning portal fibrosis on Masson-Goldner stain (magnification 50×)**.

**Figure 4:**
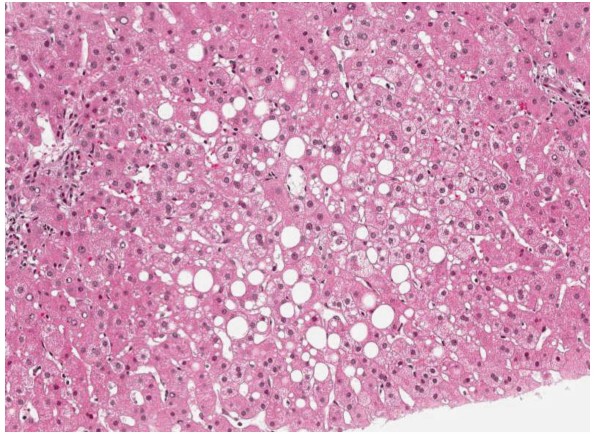
**Liver biopsy shows micro- and macrovesicular steatosis on hematoxylin-eosin stain (magnification 100×)**.

**Figure 5:**
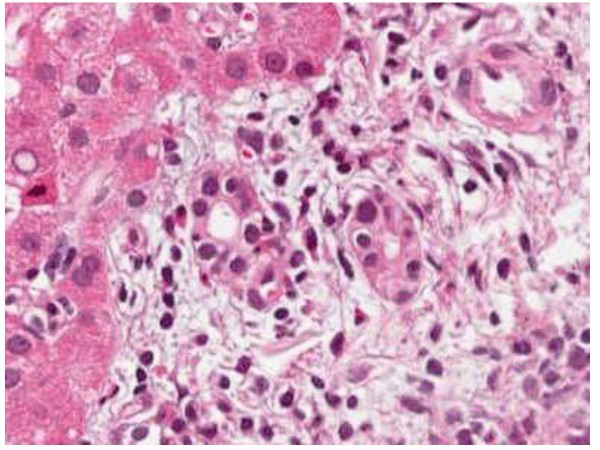
**Liver biopsy shows cholangiolitis and single cell necrosis on hematoxylin-eosin stain (magnification 200×)**.

Abdominal ultrasound demonstrated fatty liver disease, pancreas, biliary tract, and portal vein were non-accessible. Upper GI endoscopy with biopsies and rapid urease test displayed moderate chronic and moderate active Hp pangastritis. In contrast, colonoscopy was unremarkable. Hp eradication therapy was prescribed, including 1 g amoxicillin b.i.d., 500 mg clarithromycin b.i.d. and 40 mg pantoprazole b.i.d. (ZacPac™) for a duration of seven days. One day after end of treatment the patient presented with a burning sensation in his throat, side stitch, mouth dryness, dark urine, and anal pruritus. Laboratory investigation showed further increased levels for ALAT [86 U/L] and GGT [185 U/L], and stably elevated levels for ASAT [59 U/L] and bilirubin [24 μmol/L] (Figure 1). Two weeks later, the patient was admitted to the Department of Surgery because of abdominal tenderness, especially after deep liver palpation, and loss of appetite including weight loss of 3 kg (BMI 26 kg/m^2^). Laboratory investigation showed further increased levels for ASAT [73 U/L], ALAT [1351 U/L] and GGT [344 U/L], with bilirubin level remaining unchanged [21 μmol/L] (Figure 1). Since initial surgical abdominal ultrasound was suspicious of acute cholecystitis, the patient was treated with 3 g sultamicillin t.i.d. for three days. The treatment was stopped because magnetic resonance cholangiopancreatography (MRCP) could not confirm this diagnosis. The gallbladder was normal without stones, as well as intra- and extrahepatic bile ducts and pancreatic duct. There was a small cyst (with a diameter of 1.3 cm) in the head of the pancreas, but no pancreatitis, pancreatic tumor, or pancreas divisum. The patient was then transferred to the Department of Internal Medicine. History of increased alcohol consumption, viral hepatitis markers (HAV-IgM/G, Anti-Hbc, Anti-HCV), α1-antitrypsin, coeruloplasmine, ferritin, protein electrophoresis, and autoimmune markers (ANA, Anti-LKM, AMA, Anti-SLA, IgG) were all negative/normal. Liver biopsy showed mild chronic hepatitis (Figure 2) with mild portal fibrosis (Figure 3) and a 30% micro- and macrovesicular steatosis (Figure 4), consistent with drug- or toxin-induced liver injury. Histology samples were sent to Professor Lobeck (Pathology, Potsdam) for a second opinion. Besides micro- and macrovesicular steatosis (Figure 4), samples showed moderately active intrahepatic cholangitis (Figure 2) and cholangiolitis (Figure 5), likely recurrent because of already existing portal fibrosis (Figure 3) (DD cholestasis, chronic inflammatory bowel disease (CED), cholangiolitic type of drug-toxicity). There were no hints for viral or auto-immune hepatitis, nor auto-immune cholangitis. The patient was discharged from the hospital one week later because liver enzymes began to normalize. Two weeks later, laboratory investigation in our outpatient department showed decreased levels for ASAT [40 U/L], ALAT [54 U/L], and GGT [119 U/L] (Figure 1). Level of bilirubin remained unchanged [25 μmol/L]. Another month later, laboratory investigation at the office of the patient's primary care physician demonstrated further reduction of liver enzymes with the exception of bilirubin.

## Discussion

We report the case of a 64-year-old patient who presented with unclear elevation of liver enzymes. Alcohol-induced liver injury, viral hepatitis, auto-immune hepatitis, Wilson's disease, hemochromatosis, and α1-antitrypsin deficiency were unlikely according to the laboratory and histology results. Cholecysto-/choledocholithiasis could also be excluded due to normal MRCP results and normal alkaline phosphatase value. Liver biopsy was suspicious for drug- or toxin-induced liver injury. Regarding the patient's drug history it was remarkable that liver enzymes started to raise shortly after completion of Hp eradication. Since pantoprazole was not very likely the culprid, clarithromycin and amoxicillin were the drugs in suspicion. It has been known for many years that several antibiotics can cause severe hepatic injury [2]. In the case of the penicillins, the combination amoxicillin-clavulanate and the penicillinase-resistant penicillins oxacillin, (di-)cloxacillin, and flucloxacillin can cause (mainly cholestatic) hepatitis. Cephalosporins have little hepatotoxicity; ceftriaxone can cause drug-induced gallstones. The potential of erythromycin and several other macrolides to cause (usually cholestatic) hepatitis is well established. Tetracyclines can cause a syndrome mimicking acute fatty liver of pregnancy, but this complication has virtually disappeared. Quinolones seem to be able to cause cholestasis. Sulfamethoxazole/trimethoprim can cause severe hepatotoxicity, especially in patients with acquired immunodeficiency syndrome (AIDS). Finally, nitrofurantoin can cause acute cholestatic and hepatocellular reactions as well as chronic hepatitis mimicking chronic auto-immune hepatitis [2]. There are three reports indicating that intrahepatic cholestasis [3], acute liver injury and even liver failure [4,5] may be caused by amoxicillin alone. Possible drug interactions include induction of anti-coagulation effects of coumarins, inhibition of effects of contraceptives, and increase of hypersensitivity reactions in combination with allopurinol (according to patient information leaflet). Regarding clarithromycin, cholestatic liver disease [6-8] and fulminant liver failure [9] have been described even more often in the literature [10,11]. Patients with cholestatic liver disease present usually with minimal elevations of ALAT and ASAT but significant elevations of alkaline phosphatase and/or GGT. It appears that these subjects have dose-related toxicity, not a hypersensitivity reaction [8]. This phenomenon has already been described in several preclinical animal models prior to the marketing authorisation of the drug [12]. Since clarithromycin is primarily metabolized in the liver the patient information leaflet warns about administration of this drug in patients with advanced liver dysfunction. In patients with mild liver dysfunction, frequent monitoring of ASAT, ALAT, GGT, alkaline phosphatase, and bilirubin is recommended. Since clarithromycin inhibits liver enzyme CYP3A, clinicians have to be aware that plasma levels of drugs that are metabolized by this enzyme may increase. Typical drugs are antiarrythmics, carbamazepine, colchicine, digoxine, HMG-CoA reductase inhibitors, oral anticoagulants, sildenafile, tadalafile, vardenafile, theophylline, tolterodine, triazolo-benzodiazepines, and zidovudine. In addition, there are drugs like pimozide, astemizole, terfenadine, and ergotamine/dihydroergotamine that are contraindicated in combination with clarithromycin due to increased toxicity based on other mechanisms. Moreover, drugs like fluconazole and ritonavire can increase plasma level of clarithromycin, thus potentiating its side effects. However, in our case, we could not detect any typical drug interactions. We presume that clarithromycin was the main culprid for the increase in liver enzymes due to the empirical probability, although an additional adverse event of amoxicillin can not be excluded. The short course of sultamicillin administration seems to be irrelevant since liver enzyme increased prior to onset of treatment. Interestingly, the pattern of elevated liver enzymes was very unusual in this case, thus significant elevation of ALAT instead of GGT/alkaline phosphatase was the major finding. There is no good explanation for this finding. However, we speculate that preexisting fatty liver disease, respectively nonalcoholic steatohepatitis (NASH) may be a prediposition for antibiotic-induced liver injury. Steatohepatitis is characterized microscopically by hepatic fat accumulation (steatosis), mixed lobular inflammation, ballooning degeneration of hepatocytes (sometimes with identifiable Mallory bodies), glycogenated hepatocyte nuclei, and pericellular fibrosis. These are features that could also be found in the liver histology of our patient, although the "chicken wire" pattern of the pericellular fibrosis, which affects portal areas only secondarily in later stages, was not present.

## Conclusion

In summary, clinicians should be aware about the presented rare adverse event of Hp eradication treatment. In patients with pre-existing liver disease like steatohepatitis, a close look at concomitantly prescribed drugs and monitoring of ASAT, ALAT, GGT, alkaline phosphatase, and bilirubin prior and after Hp eradication is recommended. Although only moderate liver injury was detected in our case, physicians should be prepared for rapid management of acute liver failure. In addition, alternative drug combinations, such as amoxicillin + metronidazole + PPI, metronidazole + doxycycline + bismuth subcitrate + PPI, or rifabutine + levofloxacine + PPI, should be discussed for Hp eradication in these patients.

## Competing interests

The authors declare that they have no competing interests.

## Authors' contributions

MW wrote the manuscript and contributed to the mangement of the patient. CM contributed to the mangement of the patient and provided further laboratory data. HL and KW did pathology studies. All authors have read and approved the final manuscript.

## Consent

Written informed consent was obtained from the patient for publication of this case report and accompanying images. A copy of the written consent is available for review by the Editor-in-Chief of this journal.
